# Flame Retardant-Functionalized Cotton Cellulose Using Phosphonate-Based Ionic Liquids

**DOI:** 10.3390/molecules25071629

**Published:** 2020-04-02

**Authors:** Karen Al Hokayem, Roland El Hage, Lenka Svecova, Belkacem Otazaghine, Nicolas Le Moigne, Rodolphe Sonnier

**Affiliations:** 1 Polymers Composites and Hybrids(PCH), IMT Mines Alès, 6, avenue de Clavières, 30100 Alès, France; karenhokayem95@gmail.com (K.A.H.); belkacem.otazaghine@mines-ales.fr (B.O.); nicolas.le-moigne@mines-ales.fr (N.L.M.); 2 LCPM, Faculty of Sciences, Lebanese University, Campus Fanar P.O.B. 90656, Lebanon; roland_hag@ul.edu.lb; 3 Univ. Grenoble Alpes, Univ. Savoie Mont Blanc, CNRS, Grenoble INP, LEPMI, 38000 Grenoble, France; Lenka.Svecova@lepmi.grenoble-inp.fr

**Keywords:** phosphonate-based ionic liquid, flame retardancy, cellulose dissolution, regeneration

## Abstract

Cellulose from cotton fibers was functionalized through a dissolution–regeneration process with phosphonate-based ionic liquids (ILs): 1,3-dimethylimidazolium methylphosphonate [DIMIM][(MeO)(H)PO_2_] and 1-ethyl-3-methylimidazolium methylphoshonate [EMIM][(MeO)(H)PO_2_]. The chemical modification of cellulose occurred through a transesterification reaction between the methyl phosphonate function of ILs and the primary alcohol functions of cellulose. The resulting cellulose structure and the amount of grafted phosphorus were then investigated by X-ray diffraction, ICP-AES, and ¹³C and ³¹P NMR spectroscopy. Depending on the IL type and initial cotton / IL ratio in the solution, regenerated cellulose contained up to 4.5% of phosphorus. The rheological behavior of cotton cellulose/ILs solutions and the microscale fire performances of modified cellulose were studied in order to ultimately prepare flame retardant cellulosic materials. Significant improvement in the flame retardancy of regenerated cellulose was obtained with a reduction of THR values down to about 5–6 kJ/g and an increase of char up to about 35 wt%.

## 1. Introduction

Cellulose processing consists of disrupting its native hierarchical and crystalline structure by cleaving intra- and intermolecular hydrogen bonds through derivatization–dissolution or direct dissolution procedures. Dissolved (derivatized) cellulose is then regenerated in a nonsolvent that allows preparing and shaping cellulosic products as films (nano-)fibers or porous structures (i.e., aerogels) [1]. Various methods exist to improve solvent accessibility and cellulose solubility, such as steam explosion, alkalization, hydrothermal, or enzymatic treatments, etc., often leading to partial degradation of cellulose chains and lower degrees of polymerization [1]. The cellulose crystal structure generally changes from type I (in the case of native cellulose as in plant fibers, bacterial and algal cellulose) to type II after regeneration (in the case of viscose, Lyocell fibers) [2].

In order to improve the cellulose dissolution process, researchers and industrialists have investigated greener and less-destructive techniques as the direct dissolution of cellulose substrates. N-methylmorpholine N-oxide (NMMO) in its monohydrate form is efficient to dissolve a high amount of cellulose (up to 25% and typically 12–14% in technical applications) and is used at the industrial scale (i.e., in the Lyocell process), but it is not thermally stable [1]. In recent years, ionic liquids (ILs) known as green solvents, because of their low volatility and good thermal stability, have also attracted attention due to their high capacity to dissolve cellulose (typically 20%) [1,3,4,5,6,7] as well as other biopolymers such as lignin [6,8,9,10,11], zein proteins or starch [12].

**Figure 1:**
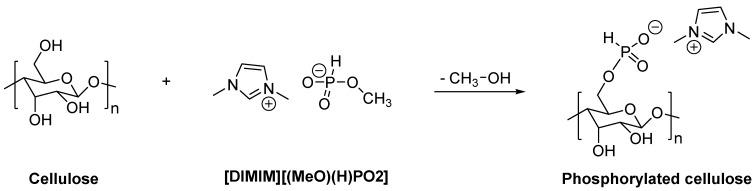
The reaction mechanism of the transesterification reaction of cellulose and [DIMIM][(MeO)(H)PO_2_], according to Vo et al. [16].

Chemical modification (i.e., derivatization) of cellulose can also be carried out using ILs as a reaction medium [4,5]. Various cellulosic materials were prepared using ILs processing [13,14,15]. Among ionic liquids, dialkylimidazolium salts with phosphonate-derived anions are a new class that can dissolve cellulose under relatively mild conditions [16,17], besides, phosphorus is known to be a key element in flame retardancy. Even though phosphonate-based ILs release a high amount of heat during burning [18,19], flame retarded materials like epoxy resins [20,21], cotton fabrics [22], or thermoplastics derived from cell wall biopolymers [23] have already been prepared using such ionic liquids. Indeed, phosphorus flame retardants are especially efficient for materials rich in hydroxyl groups. During the dissolution of cellulose using a phosphonate-based IL (namely [DIMIM][(MeO)(H)PO_2_]), a reaction between hydroxyl groups of cellulose and the phosphonate function of IL can occur [16]. This reaction leads to the phosphorylation of the primary alcohol (C6) of cellulose (Figure 1). Moreover, thermogravimetric analysis showed a significant increase in char formation of the modified cellulose compared to pure cellulose. Nevertheless, the flame retardancy of this modified cellulose was not investigated.

Based on these findings, the objective of the present study was to investigate the functionalization of cotton cellulose through a direct dissolution–regeneration procedure using two phosphonate-based ILs (namely 1,3-dimethylimidazolium methylphosphonate [DIMIM][(MeO)(H)PO_2_] and 1-ethyl-3-methylimidazolium methylphosphonate [EMIM][(MeO)(H)PO_2_]) and its flame retardancy after regeneration. The ultimate objective was to prepare cellulosic materials exhibiting high fire performances. With this regard, the rheological behavior of cotton cellulose/ILs solutions was studied as it is a key parameter for shaping cellulose products from solutions.

## 2. Results

### 2.1. Dissolution and Regeneration of Cotton Cellulose/ILs

**Figure 2:**
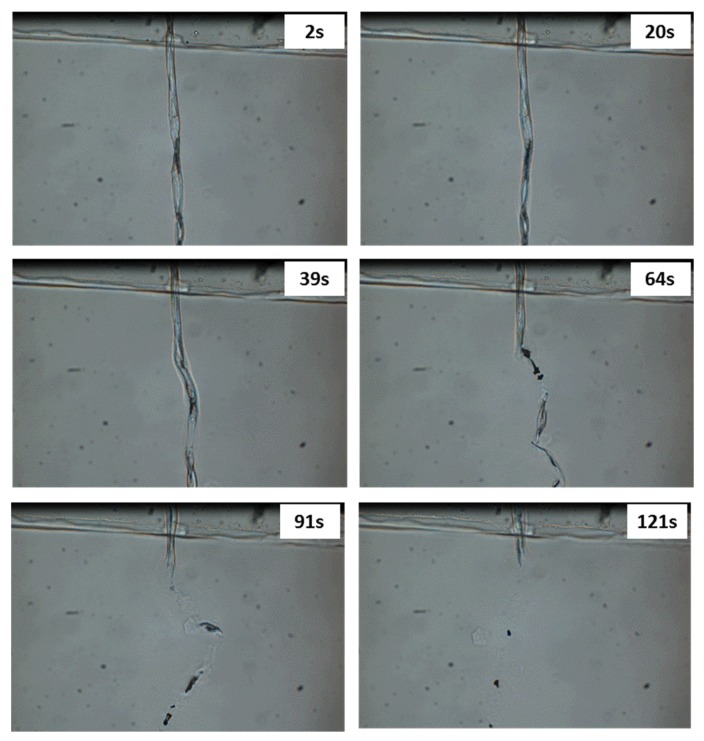
Optical microscopy pictures of the dissolution of a single cotton fiber in [DIMIM][(MeO)(H)PO_2_] at 80 °C.

The dissolution behavior of cotton fibers in different ILs was firstly studied by optical microscopy, to attest their effective dissolution in a large excess of ILs. Figure 2 shows pictures of a single cotton fiber in [DIMIM][(MeO)(H)PO_2_] at 80 °C at different dissolution times. Full dissolution became obvious a few dozen seconds after the beginning of the test and was complete in 2 min, evidencing the high ability of the tested IL to dissolve cellulose even at a temperature lower than 100 °C. The same observation was made with [EMIM][(MeO)(H)PO_2_] and [Bmim] [OAc].

**Figure 3:**
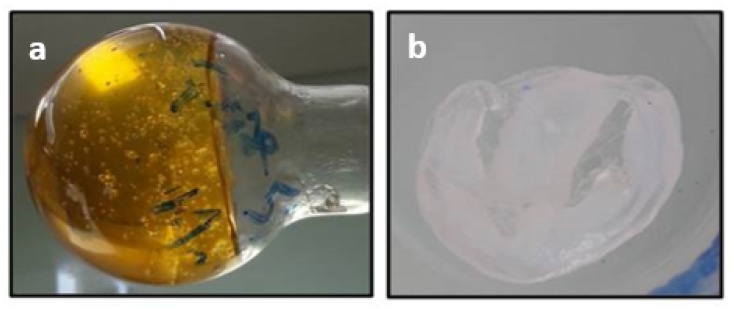
Photographs of: (**a**) dissolved cotton cellulose in [DIMIM][(MeO)(H)PO_2_] (5 wt%); (**b**) cellulose regenerated in ethanol.

Raw cotton fibers were then dissolved at various concentrations ranging from 0.5 wt% to 20 wt% in ILs at 100 °C. Figure 3a,b shows photographs of the dissolved cotton in [DIMIM][(MeO)(H)PO_2_] and the cellulose regenerated in ethanol, respectively.

When cotton fraction was ranged from 0.5 wt% to 10 wt%, complete dissolution (no visible insolubles) was observed for [BMIM][OAc] (used for a comparative purpose), but also for both ILs containing phosphorus. For a mass fraction of 20 wt%, dissolution was incomplete (many undissolved fibers visible in the solution). This phenomenon is explained by the important viscosity of the solution and stirring energy, which was insufficient to homogenize the suspension properly. Furthermore, it is known that 20 wt% of cellulose in solution is close to its solubility limit in ILs [7]. After regeneration in ethanol and drying, no weight gain was noticed when [BMIM][OAc] was used. On the contrary, a significant weight gain was measured for the regenerated cellulose dissolved using both phosphonate-based ILs. As observed in Table 1, the weight gain depends on the initial mass fraction of cotton in IL. The value of the weight gain for the cellulose sample after regeneration decreased when the cotton/IL ratio increased. Moreover, for the same cotton concentration, the weight gain was slightly higher for [DIMIM][(MeO)(H)PO_2_] than [EMIM][(MeO)(H)PO_2_].

### 2.2. Characterization of Regenerated Cellulose

**Figure 4:**
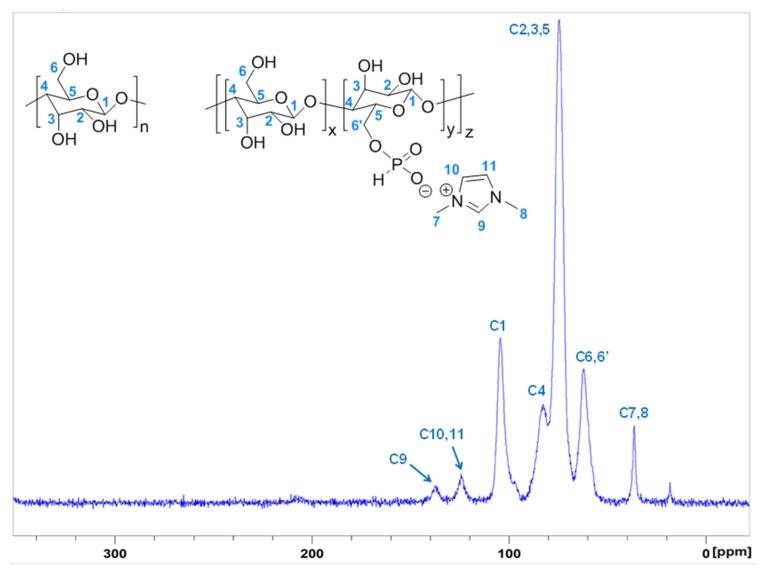
¹³C NMR spectrum of regenerated cellulose dissolved in [DIMIM][(MeO)(H)PO_2_] (mass fraction of cotton fiber in [DIMIM][(MeO)(H)PO_2_]; 5 wt%).

**Figure 5:**
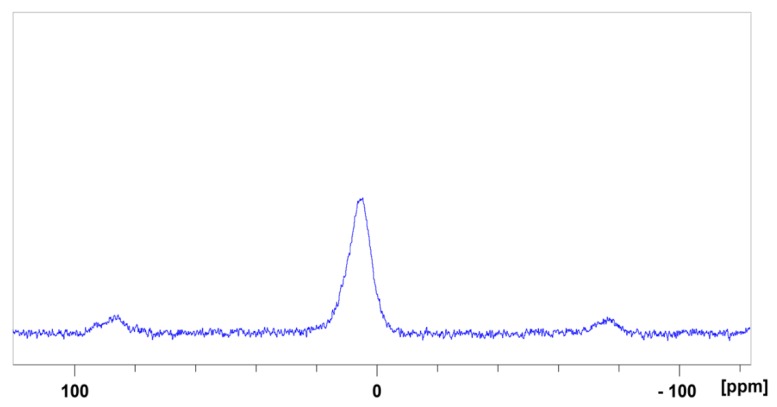
³¹P NMR spectrum of regenerated cellulose dissolved in [DIMIM][(MeO)(H)PO_2_] (mass fraction of cotton in [DIMIM][(MeO)(H)PO_2_]; 5 wt%).

Cellulose modified by the dissolution in [DIMIM][(MeO)(H)PO_2_] and regenerated by ethanol addition was analyzed by ^13^C and ^31^P solid-state NMR. The ¹³C NMR spectrum (Figure 4) was in agreement with the results obtained by Vo et al. [16]. As observed by these authors, in addition to characteristic peaks of carbon atoms of the anhydroglucose units (AGU) of cellulose, new peaks appeared at 38, 125, and 140 ppm assigned to imidazolium group. The presence of these signals may prove the modification of some AGU units by the transesterification reaction between some primary hydroxyl groups of cellulose and the phosphonate-based ILs. ³¹P NMR spectrum also confirms the presence of phosphorus for regenerated cellulose (Figure 5). The peak observed at 4.6 ppm was attributed to the phosphonate group of the IL that may be grafted to cellulose. According to Figure 1, an ionic structure based on the reaction of the IL solvent and AGU of cellulose (i.e., with a negative charge on the phosphonated group and a positive charge of the imidazolium cation), was obtained.

Taking into account the fact that both imidazolium and phosphonate moieties have been identified to be present on modified cellulose, the weight increase was undoubtedly attributable to both the anion and the cation. According to Figure 1, the phosphorus content can be deduced from the sample weight increase (Table 1) after the dissolution–regeneration procedure, considering the molecular weight of IL minus the weight of the group –OCH_3_. The P content was also determined more accurately using the ICP-AES technique after the prior dissolution of the solid. Table 1 lists the phosphorus contents in the various phosphorylated cellulose samples obtained from weighting and ICP-AES analysis. It can be seen that the use of [DIMIM][(MeO)(H)PO_2_] leads to higher phosphorus content compared to [EMIM][(MeO)(H)PO_2_]. It should be noted that based on ICP-AES results, weighting slightly overestimates the phosphorus content. Finally, the phosphorus content tends to decrease when the content of cotton fibers in ILs increases. Nevertheless, in all cases, the phosphorus content remains higher than 1 wt% (and can be as high as 5 wt%). Such contents should provide appreciable flame retardancy to the regenerated cellulose. All these results confirmed that cellulose and phosphonate-based ILs interact and are indicative of the phosphorylation of the regenerated cellulose. It should also be mentioned that Vo et al. obtained higher P contents ranging from 5% to 9.5% [16]. However, slightly different experimental conditions have were used in their study (temperature ranging from 120 to 160 °C, and reaction time ranging from 1 to 3 h). These differences may explain the differences in the results obtained.

The degree of substitution DS, i.e., the number of substituent units per glucose unit, was also calculated according to equation 1, considering the phosphorus contents measured by ICP-AES and the nature of substituent determined from NMR analyses. DS was found to range from 0.12 to 0.31 for cellulose modified by [DIMIM][(MeO)(H)PO_2_] (corresponding to 2.02 wt% and 4.51 wt% of phosphorus respectively). DS was only 0.07 (1.31 wt% of phosphorus) for cellulose modified by [EMIM][(MeO)(H)PO_2_], while there were three hydroxyl groups per glucose unit, meaning that only a few of them were substituted;

(1) $$\mathrm{DS}=\frac{f_{S}\times M_{\mathrm{glucose}}}{M_{S}-f_{S}\times \left(M_{S}-1\right)}$$

with f_S_ the mass fraction of the substituent, M_glucose_ the molar mass of the glucose unit, M_S_ the molar mass of the substituent.

It should be noted that Hirosawa et al. have investigated in detail the solvated structure of cellulose dissolved in [EMIM][(MeO)(H)PO_2_] [24]. Based on high-energy X-ray total scattering experiments and molecular dynamics simulations, the authors assumed that cellulose was dispersed at the molecular level in this IL but negatively charged oxygen atoms in IL anion species only formed hydrogen bonds with hydroxyl groups of cellulose. Accordingly, in the dissolved state, cellulose was thus not phosphorylated, i.e., grafting of anion species onto cellulose hydroxyls. Note that the dissolution was performed at low temperatures in this work (50 °C versus 100 °C in our work and 120 °C in the work of Vo et al.). Furthermore, Hirosawa et al. [24] analyzed cellulose/ILs interactions in the dissolved state and not in the solid and crystallized state, as was the case in our study.

However, it is true that our ^13^C NMR results do not prove the transesterification reaction due to possible overlapping of peaks. Nevertheless, the presence of these peaks, even after the washing step (Soxhlet 12 h at around 80 °C) and the results obtained by Vo et al., suggest that part of the cellulose was phosphorylated.

**Figure 6:**
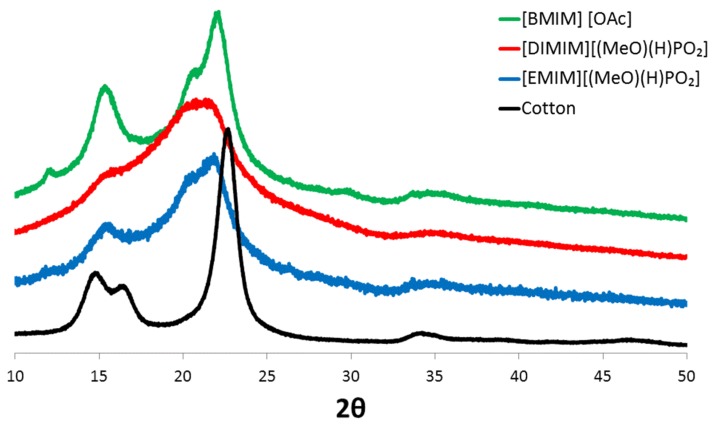
XRD spectra of raw cotton and cellulose dissolved and regenerated using [DIMIM][(MeO)(H)PO_2_], [EMIM][(MeO)(H)PO_2_] and [BMIM] [OAc] (cotton fiber content in ionic liquids = 5 wt% for phosphonate-based ILs and 10 wt% for [BMIM] [OAc]).

Additionally, the crystalline structure of the modified cellulose was compared to those of the initial cotton cellulose using X-ray diffraction. The respective spectra of raw cotton and phosphorylated cellulose are shown in Figure 6. A typical pattern of Cellulose I was observed for raw cotton with the main peaks at 14.7°, 16.4°, 22.7°, and 34.1° corresponding respectively to crystallographic planes (11 0), (110), (200), and (004) [25,26,27]. After dissolution in the ILs and regeneration, XRD spectra are significantly different. The peak at 22.7° was shifted to 21.5–21.8°. The shoulder at 20.5° was assigned to the shift of the peak associated with the (110) plane [25]. A very small peak at 12° for regenerated cellulose modified with [EMIM][(MeO)(H)PO_2_] was assigned to the shift of the peak associated with the (11 0) plane. Regenerated cellulose dissolved with [BMIM] [OAc] also exhibited these changes, which revealed the transition from Cellulose I to Cellulose II during dissolution–regeneration, as already stated by many authors. Nevertheless, the peaks are much less defined for cellulose dissolved by phosphonate-based ILs and regenerated, revealing the loss of highly ordered crystalline areas.

It can thus be assumed that the phosphorylation of cellulose impedes the organization of cellulose chains in well-ordered crystalline structure upon regeneration, possibly due to the steric hindrance of the substituent, hence resulting in less crystalline regenerated cellulose.

### 2.3. Flammability of Regenerated Cellulose

At the microscale, phosphorus flame retarded lignocellulosic materials present increasing char content, decreasing peaks of heat release rate (HRR), and total heat release (THR), but also decreasing thermal stability compared to their nontreated homologs. Indeed, when temperature increases, the phosphorus flame retardant decomposes, leading to the formation of phosphoric acid, which can phosphorylate cellulose and promote the formation of phosphorus esters, which accelerate dehydration of cellulose enhancing the formation of char [28]. Thus, charring is promoted by the presence of phosphorus, leading to higher residue yield and lower THR, which is obviously considered as a positive result. Moreover, a quite good correlation was found between PCFC results and a lab-made fire test assessing the self-extinguishment of phosphorus molecules grafted flax fabrics when ignited at the top of samples in a vertical position [29]; self-extinguishment was especially systematically observed when THR was below approximately 5 kJ/g. Obviously, flax composition and structure are different from those of the phosphorylated cellulose, but these previous observations can be considered as a guideline to assess the flame retardancy of our materials.

**Figure 7:**
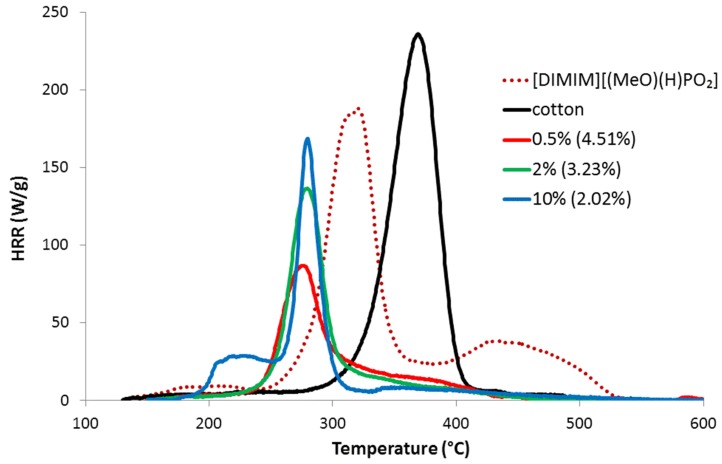
HRR versus temperature curves for [DIMIM][(MeO)(H)PO_2_], raw cotton and for regenerated cellulose obtained from the dissolution of various contents of cotton into [DIMIM][(MeO)(H)PO_2_] (in brackets, phosphorus contents from ICP-AES).

Figure 7 shows some HRR curves for pure cotton and regenerated cellulose dissolved in [DIMIM][(MeO)(H)PO_2_]; similar results were obtained for cellulose dissolved in [EMIM][(MeO)(H)PO_2_]. The HRR curves exhibited the typical tendencies observed for phosphorylated lignocellulosic materials: a decrease in the peak of heat release rate (pHRR) and a lower THR (i.e., the area under the HRR curve), however, lower thermal stability was also observed. Interestingly, an additional peak at low temperature can be observed, but only for the cellulose sample prepared by dissolution of 10 wt% of cotton in the IL; this peak did not correspond to pure IL (compare with HRR curve for pure [DIMIM][(MeO)(H)PO_2_]) and remains unexplained currently.

**Figure 8:**
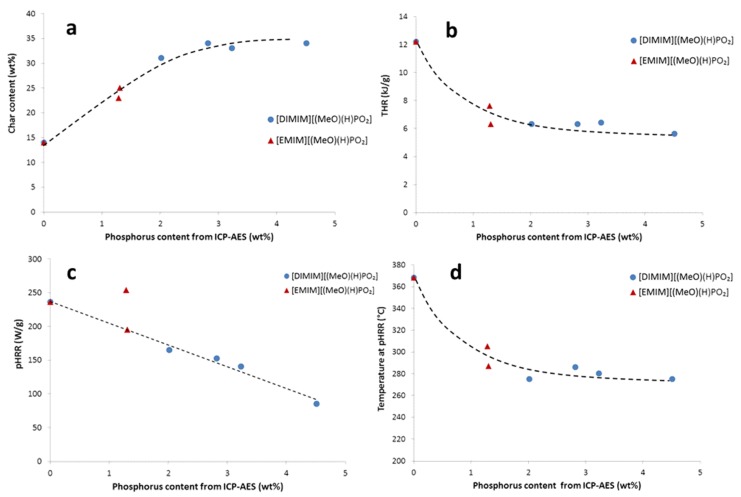
The influence of phosphorus content in the modified cellulose on (**a**) char content, (**b**) THR, (**c**) pHRR, and (**d**) temperature at pHRR from PCFC analysis.

Figure 8 summarizes the evolution of the main PCFC data as a function of increasing phosphorus content of the regenerated cellulose samples obtained with both phosphonate-based ILs. For the phosphorus molecules grafted flax fabrics, the char content (Figure 8a) increased systematically from less than 15 wt% to 30–35 wt%. Concomitantly, THR decreased because carbon was partially trapped into the condensed phase (Figure 8b). The heat of combustion (calculated as THR divided by mass loss fraction) was approximately 14 kJ/g for cotton and 6–10 kJ/g for the modified cellulose. Moreover, the heat of combustion tended to decrease when phosphorus content increased. pHRR also decreased from more than 200 W/g to around 80 W/g when phosphorus content increased (Figure 8c). As stated, phosphorus promotes earlier decomposition of cellulose, which explains why the temperature of pHRR decreased from 370 °C to 280–260 °C when phosphorus content was superior to 2 wt% (Figure 8d). The evolution of PCFC data was thus closely correlated to the phosphorus content. There is no significant difference between samples treated either by [DIMIM][(MeO)(H)PO_2_] or [EMIM][(MeO)(H)PO_2_] at similar phosphorus content.

**Figure 9:**
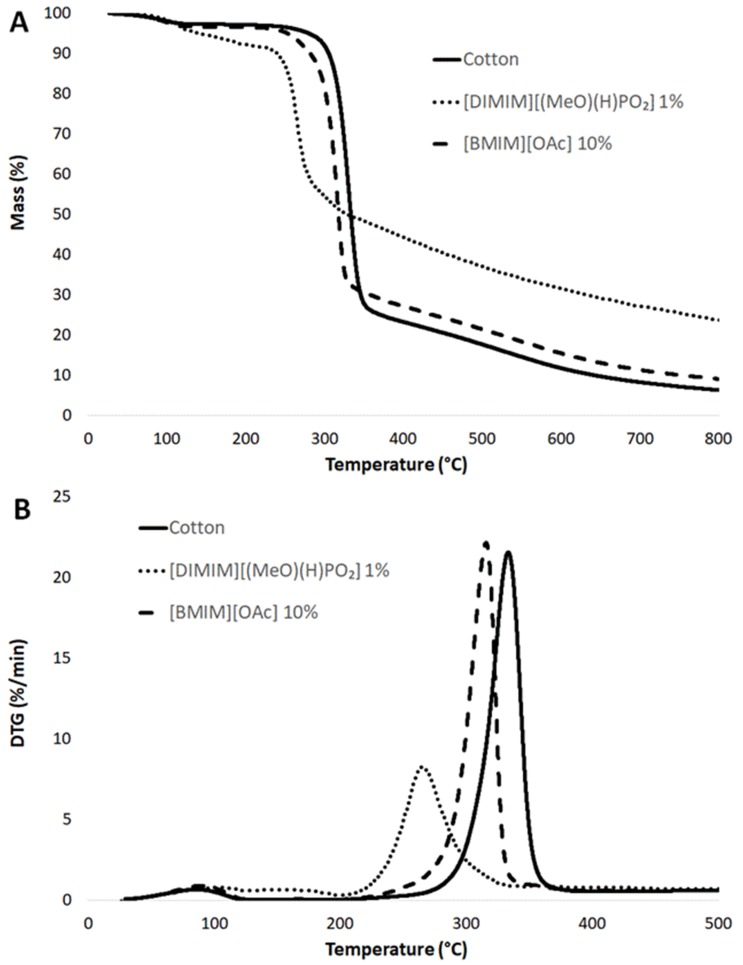
Thermogravimetric curves of raw cotton and regenerated cellulose samples obtained, respectively, by the dissolution of 1 wt% cotton in [DIMIM][(MeO)(H)PO_2_] at 100 °C and 10 wt% cotton in [BMIM] [OAc] at 100 °C. (**A**) Thermogravimetric curves, (**B**) Derivative thermogravimetric curves.

Thermogravimetric analyses were also performed (Figure 9). All samples exhibited a limited mass loss (of around 3 wt%) at around 100 °C. This first mass loss seemed to be higher (8 wt%) and took place over a wider range of temperatures for the phosphorylated cellulose (4 wt% of phosphorus according to Table 1), confirming that phosphorylated cellulose was more hydrophilic, as indicated by Vo et al. [16]. The decomposition curve of regenerated cellulose dissolved in [BMIM] [OAc] was very similar to the curve for raw cotton (the temperature at maximum loss rate was 333 °C and residue content at 700 °C was 8 wt%). Only a slight shift of the main decomposition step (−15 °C as compared to raw cotton) and a slight increase of char residue (11 wt% at 700 °C) were observed. This result evidenced that the modification of the cellulose structure due to the dissolution–regeneration process (as discussed from XRD spectra in Figure 6) did not modify the decomposition pathway of cellulose significantly. On the contrary, the decomposition curve of phosphorylated cellulose was strongly modified. The main decomposition step occured at a lower temperature (−55 °C as compared to raw cotton) but its intensity was reduced. Moreover, the char content was much higher (27 wt% versus only 8 wt% for raw cotton at 700 °C). It can be noted that this char was not completely stable and the mass loss occurred slowly up to 800 °C. All these results (lower thermal stability, lower intensity of the main decomposition step, and higher char content) were in agreement with the previous PCFC analyses.

All the results are qualitatively in good agreement with previous results related to the effect of phosphorus on the thermal stability and fire retardancy of phosphorus modified (ligno-)cellulosic substrates. Using thermogravimetric analysis, Vo et al. recorded high char contents (up to more than 40 wt% at 500 °C) for phosphorylated cellulose obtained by dissolution–regeneration [16]. Hajj et al. also observed that the flame retardancy of phosphorus-based monomer grafted flax at microscale does not depend on the structure of the phosphorus-based monomer but only on phosphorus content [29]. The thermal stability of treated flax was also strongly decreased up to 260 °C (against about 360 °C for raw flax) for 1–2 wt% of phosphorus. Nevertheless, the authors found that char content reached 30–35 wt% while THR and pHRR decreased below 3–4 kJ/g and 50 W/g respectively for phosphorus content in the range 1–2 wt%. In other words, while all PCFC characteristics were very similar for raw flax and cotton fibers, grafting phosphorus led to better flame retardancy in the case of flax (for the same phosphorus content). However, some differences can be pointed out between both studies: mainly the nature of flame retardant (phosphonate-based ionic liquid versus various phosphonates and phosphonic acids) but also the modification procedure (monomer grafting for modified flax versus dissolution–regeneration for cotton) and the nature and structure of bio-resource (flax fibers versus regenerated cellulose). Nevertheless, the relatively lower efficiency of phosphorus to improve flame retardancy for regenerated cellulose as compared to flax fibers deserves further investigation.

Regardless of the effect of phosphorus, it was also mentioned in the literature that the char content was also dependent on the cellulose fine structure. A high degree of cellulose crystallinity promotes the formation of levoglucosan during pyrolysis and enhances flammability [30]. Besides, Swatloski et al. observed lower thermal stability but a considerable enhancement of char content after regeneration of cellulose using a phosphorus-free IL ([C_4_mim]Cl) [31]; therefore, a lower degree of crystallinity and higher phosphorus content may both contribute to lower the flammability of cellulose. Nevertheless, both phenomena are related. First, crystallization upon regeneration is prevented by phosphorylation, as proven by XRD analyses, and second, Basch and Lewin have shown that the efficiency of phosphorus flame retardants depends on the fine structure of cellulose [32].

To discriminate the influence of these parameters, flammability of regenerated cellulose after dissolution in [BMIM] [OAc] (mass fraction of cotton in IL; 10 wt%) was studied. Note that this regenerated cellulose is less crystalline than raw cotton but higher than phosphorylated cellulose, according to XRD analyses (Figure 6). For this regenerated cellulose, the pHRR was measured at 175 W/g, at a temperature of 345 °C, while THR was around 9 kJ/g. Char content remained as low as 14 wt%. Regenerated cellulose after dissolution in [BMIM] [OAc] has thus had slightly lower flammability than raw cotton with a pHRR higher than 200 W/g and a THR equal to 12 kJ/g, which could be related to its less crystalline structure. Nevertheless, the flammability properties remained worse than those determined for phosphorylated cellulose; char especially was not significantly promoted when cellulose was regenerated after the dissolution in [BMIM] [OAc].

All these results evidence that the phosphorylation of cellulose by dissolution–regeneration with phosphorus-containing ILs leads to better flame retardancy properties, mainly because of the presence of phosphorus as a char promoter, the amorphization of cellulose (induced by the grafting of phosphorus IL) has a more limited influence. Finally, even if a fraction of ionic liquid is only physically trapped in cellulose (and not covalently grafted), this would not be an issue for potential applications. Indeed, TGA and PCFC analyses show that the interactions between the phosphorous compound and cellulose are strong enough to improve its flame retardancy and to avoid a premature release of phosphorus compound during processing steps or heating.

### 2.4. Processing of Regenerated Cellulose

**Figure 10:**
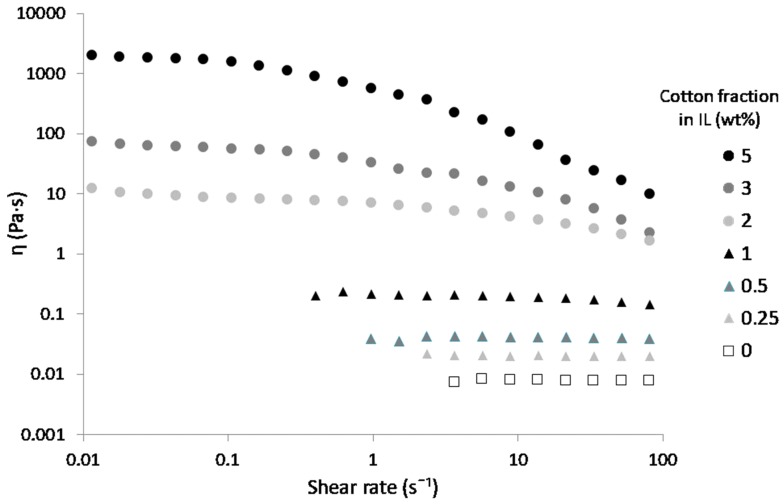
Shear viscosity η versus the shear rate at 100 °C for solutions of [DIMIM][(MeO)(H)PO_2_] containing various amounts of cotton.

The modified and flame retarded cellulose may be valuable to prepare new cellulosic materials, but the rheological behavior of cellulose/ILs mixtures is critical for their further processing and shaping into cellulose products. In this part, only [DIMIM][(MeO)(H)PO_2_] was used because it allowed the highest phosphorus content to be reached. Figure 10 shows the shear viscosity versus shear rate curves for solutions of [DIMIM][(MeO)(H)PO_2_] containing various contents of cellulose. A Newtonian behavior was observed up to 1 wt% of dissolved cotton fibers in IL. A shear-thinning behavior at shear rates higher than 0.1 s^−1^ was observed for higher cotton fiber contents, i.e., from 2 to 5 wt%.

**Figure 11:**
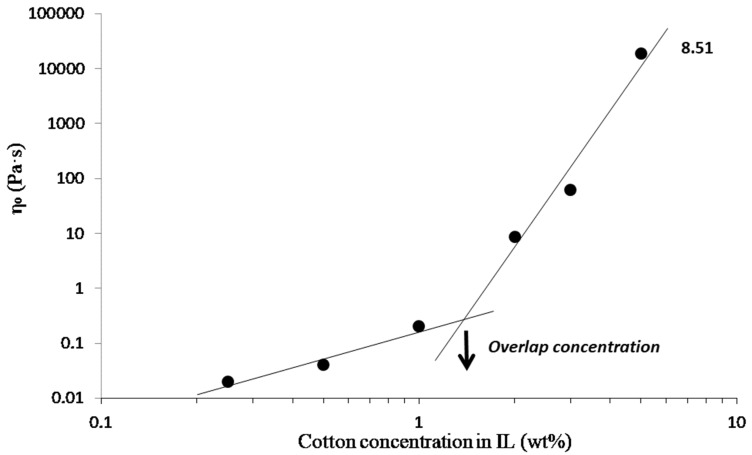
The influence of cotton’s fiber concentration in [DIMIM][(MeO)(H)PO_2_] on zero shear viscosity η_0_ of solution at 100 °C. Lines are power-law approximations, and the power-law exponent is given for the semi-dilute regime.

From these viscosity curves, the zero shear viscosity η_0_ was taken from the viscosity plateau obtained for each cellulose/IL solution. Plotting η_0_ versus the mass fraction of cotton fiber in the IL (Figure 11) allowed the identification of two regions: a diluted regime and a semi-dilute one (power-law ~ C^8.51^) above the overlap concentration C* [33]. Below this concentration, the viscosity was low (0.02 Pa·s for 0.25 wt% of cotton fiber in IL), and interactions between cellulose chains in the solution were limited. Viscosity increased drastically above the overlap concentration to reach 18,600 Pa·s for 5 wt% of cotton fibers in IL, which was indicative of entanglements and strong interactions between cellulose chains in the solution. The overlap concentration C* from diluted to the semi-dilute regime was reached at roughly 1.45 wt% of cotton fibers in IL for [DIMIM][(MeO)(H)PO_2_]. This value of C* is in agreement with results obtained by Gericke et al. [34] and Sescousse et al. [35] with cellulose of various degrees of polymerization (DP) dissolved in [EMIM][OAc]. Note that the power-law exponent is significantly higher in the semi-dilute regime what might originate from the high DP of cotton cellulose and/or specific intermolecular interactions in the presence of [DIMIM][(MeO)(H)PO_2_].

**Figure 12:**
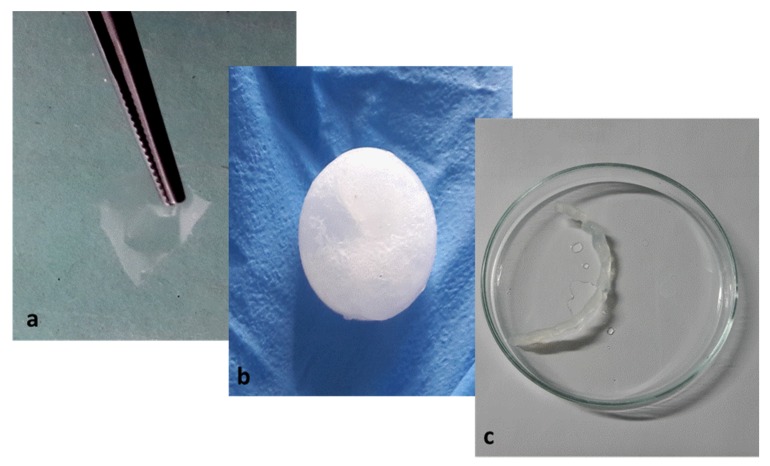
Examples of objects based on phosphorylated regenerated cellulose: (**a**) regenerated film from 2 wt% cotton fibers in [DIMIM][(MeO)(H)PO_2_], (**b**) regenerated bead, and (**c**) regenerated yarn from 5 wt% cotton fibers in [DIMIM][(MeO)(H)PO_2_].

The shaping of cellulosic products is usually achieved with concentrated cellulose solutions, i.e., above the overlap concentration, where zero shear viscosities reach several thousand Pa·s [36]. The strength of the cellulose solution is, indeed, critical in processes involving extension flow and high shearing, such as filming or fiber spinning [37]. Different objects have been prepared from the regenerated cellulose, i.e., film, beads, and yarn (Figure 12). These objects have been produced using the cotton fibers/[DIMIM][(MeO)(H)PO_2_] solution above the overlap concentration, i.e., 1.45 wt%. Based on our results, 2 to 5 wt% cotton fiber solutions were considered suitable for classical cellulose processing. The continuous thin film and the yarn are transparent and resistant to stretching. The pellet is white and hard. As expected, the regeneration step in ethanol leads to a strong shrinkage of the objects, which needs further optimization, in particular by controlling the cellulose content in the solution and the nature of the regeneration bath. However, these first attempts prove that it is possible to easily prepare various objects from phosphorylated cellulose.

**Figure 13:**
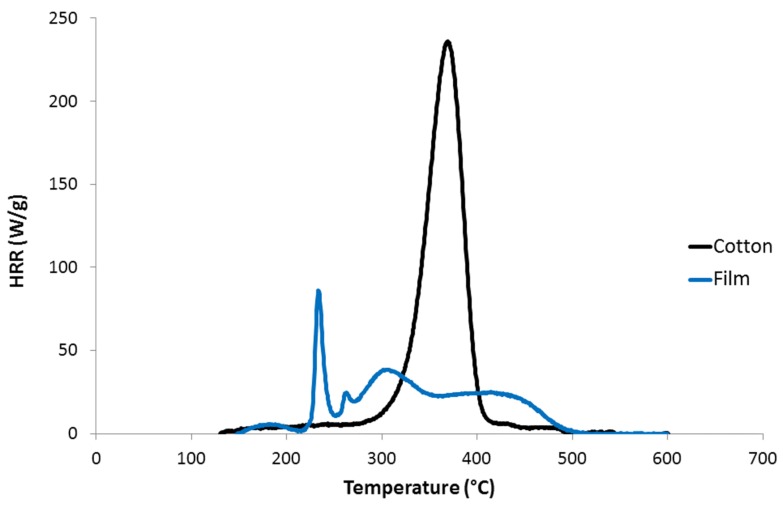
HRR versus temperature curves for cotton fibers and regenerated film prepared from a solution of [DIMIM][(MeO)(H)PO_2_] containing 2 wt% of cotton fibers.

PCFC analysis was also carried out on the cellulosic film prepared from [DIMIM][(MeO)(H)PO_2_] containing 2 wt% of cotton fibers (Figure 13). The main pHRR of the film was very low (<100 W/g) at 235 °C, while THR was reduced to 6.8 kJ/g, and char was increased to 26 wt%, confirming the results discussed above. Two other HRR peaks can be observed and correspond to the main decomposition steps of the IL (compare with HRR curve for [DIMIM][(MeO)(H)PO_2_] in Figure 7). These peaks could correspond to the decomposition of imidazolium cation or to a fraction of IL, which was not fully removed during regeneration in ethanol bath. A deeper analysis of the kinetics of cellulose regeneration and diffusion of IL in ethanol would be interesting to improve the regeneration process and avoid residual IL in regenerated cellulose.

## 3. Materials and Methods

### 3.1. Materials

The cotton used within this work was 100% hydrophilic, bleached and pre-cut commercial pure cotton, available in French supermarkets, and having a “Leader Price” trademark. Absolute ethanol was purchased from Fisher Scientific (Waltham, MA, USA) and used as received.

**Figure 14:**
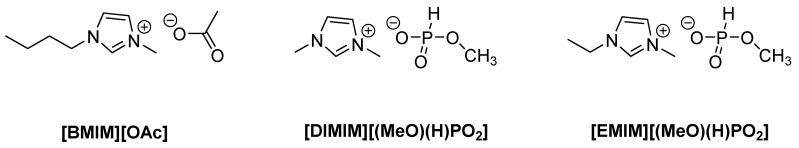
Chemical structures of the used ILs.

1-Butyl-3-methylimidazolium acetate ([BMIM][OAc]) was purchased from Iolitec (Heilbronn, Germany). 1,3-Dimethylimidazolium methyl-phosphonate and 1-ethyl-3-methylimidazolium methyl-phosphonate (respectively [DIMIM][(MeO)(H)PO_2_] and [EMIM][(MeO)(H)PO_2_]]) (Figure 14) were purchased from Solvionic SA (Toulouse, France). All ILs used were liquid at room temperature, with purity superior to 98%.

### 3.2. Experimental and Characterization Procedures

The dissolution behavior of individual cotton fibers in the three studied ILs was monitored by optical microscopy in transmission mode with a Laborlux 12 Pol S (Leica, Wetzlar, Germany) equipped with a mono-CDD Sony digital camera (resolution 1600 × 1200 pixels, Tokyo, Japan) and an image acquisition software Archimed^®^ (Microvision Instruments, Lisses, France). One cotton fiber was maintained between two glass plates and positioned on a hot stage at 80 °C. The IL was preheated at 80 °C and injected by capillarity between the two glass plates using a pipette. No agitation was applied to the system.

Phosphorylation of cellulose was achieved by dissolving various amounts of cotton in 1 g of IL with vigorous magnetic stirring in a one-necked round-bottom flask (50 mL) closed by a cap at 100 °C for 5 h. Ethanol was then added in excess to regenerate the dissolved cellulose. The resulting modified cellulose was filtered and washed with ethanol using a Soxhlet apparatus during 12 h. Finally, the modified cellulose is dried in an oven for 5 h at 80 °C. Note that this dissolution–regeneration procedure involved chemical modification of cellulose.

The evolution of cellulose weight before and after the dissolution was monitored by weighing. The phosphorus content in the modified cellulose was also measured by inductively coupled plasma atomic emission spectroscopy (ICP-AES; HORIBA Jobin Yvon; Activa M, Kyoto, Japan). Prior to the ICP-AES analyses, the samples were mineralized using MILESTONE 1200-MEGA digestion microwave using HNO_3_ (63%) and H_2_SO_4_ (98%) mixture.

Cross-polarization and magic angle rotation (CP/MAS) of ¹³C and ^31^P NMR analyses at room temperature were carried out using a Bruker Avance-300 spectrometer operating at a frequency of 100.59 MHz (Bruker, Billerica, MA, USA).

XRD patterns were obtained using a Bruker AXS D8 advance diffractometer with CuKα radiation and a Vantec detector. The scanning range was from 5° to 50°, with a step size of 0.007°.

A pyrolysis combustion flow calorimeter (PCFC; Fire Testing Technology Ltd., East Grinstead, UK) was used to investigate the fire behavior of samples at microscale (2–4 mg). Sample pyrolysis was performed at a heating rate of 1 °C/s under nitrogen flow (100 mL/min) from 90 to 750 °C (anaerobic pyrolysis method A, according to the standard ASTM D7309). Pyrolysis gases were carried to a combustor in the presence of an N_2_/O_2_ (80/20) mixture. In such conditions, all gases were fully oxidized. Heat rate release (HRR) was calculated according to Huggett’s relation [38], which states that 1 kg of consumed oxygen corresponds to 13.1 MJ of heat release. Each test on a single sample was performed twice to ensure the reproducibility of our measurements. The peak of heat rate release (pHRR), the temperature at pHRR (Tmax), and the total heat release (THR) were determined.

Thermogravimetric analyses (TGA) were performed using Setaram Setsys apparatus (Setaram, Caluire-et-Cuire, France). Samples (few mg) were heated at 10 °C/min under nitrogen flow (anaerobic pyrolysis).

The rheological behavior of the dissolved cellulose solutions in [DIMIM][(MeO)(H)PO_2_] IL was evaluated using a TA Instruments AR–2000 N cone/plate rheometer (TA Instruments, New Castle, DE, USA) having a 40 mm diameter and 4° cone on different prepared solutions with various concentrations, 0.25, 0.5, 1, 2, 3, and 5 wt% of cellulose in IL. All measurements were carried at 100 °C out using 2 mL solutions by applying a shear rate sweep from 10^−3^ to 100 s^−1^.

### 3.3. The Shaping of Regenerated Cellulose Objects

The viscous solution of 5 %wt of cotton dissolved in [DIMIM][(MeO)(H)PO_2_] was used to prepare a pellet and a yarn of regenerated cellulose. Due to its high viscosity and after applying pressure, this solution took the form of the container in which it was placed. Two different objects were prepared: a pellet using a ring and a yarn using a syringe.

An excess of ethanol was then added to precipitate the dissolved cellulose and remove the ionic liquid. The pellet and the yarn were also washed overnight with an ethanol Soxhlet to remove the residual ionic liquid. This washing step was essential and led to the improvement of the fire behavior of regenerated cellulose objects. Finally, the regenerated cellulose pellet and yarn were dried in an oven at 80 °C for 5 h.

A thin film of regenerated cellulose was formed with a solution of 2 %wt of cotton dissolved in [DIMIM][(MeO)(H)PO_2_]. The surface of a thin aluminum plate was covered by a sufficient quantity of this solution using a spatula. After that, the plate was immersed in a crystallizer containing ethanol to regenerate the cellulose and remove the ionic liquid. Finally, the plate was removed and dried under the hood.

## 4. Conclusions

In order to prepare flame retarded cellulosic materials, cotton fibers were dissolved using two different phosphonate-based ILs, i.e., [DIMIM][(MeO)(H)PO_2_] and [EMIM][(MeO)(H)PO_2_]. Regeneration of modified cellulose was achieved by Soxhlet extraction in ethanol. It was shown by weighing and ICP-AES measurements that a fraction of ionic liquids remained in the regenerated cellulose. As attested by ^13^C and ^31^P solid-state NMR, grafting reaction could occur during the dissolution procedure between primary hydroxyl groups of AGU units of cellulose and phosphonate group of phosphonate-based ILs. Cellulose modification also substantially modified the crystalline structure of regenerated cellulose, which was found to be less crystalline.

The final phosphorus content of regenerated cellulose depends on the cotton/IL ratio used for the dissolution procedure and can reach up to 4.5 wt% according to ICP measurements. Flammability of modified cellulose is significantly reduced with the decrease of heat release and promotion of char when phosphorus content increases. Ultimate reduction of THR down to about 5–6 kJ/g and increase of char up to 35 wt% were obtained for 4 wt% of phosphorus. It is noteworthy that the amorphization of regenerated cellulose may also contribute to lowering its flammability.

Various objects, i.e., film, bead and yarn, have been easily prepared from the IL/cellulose solutions. Even if such solutions are easily shapeable, shrinkage and residual IL after regeneration are major issues and further investigations are needed to overcome it, in particular studying the effect of cellulose content in the solution, the nature of the regeneration bath and kinetics of cellulose regeneration should be investigated in future works.

## Figures and Tables

**Table 1:** The influence of fiber concentration on the weight gain and phosphorus content after the dissolution and regeneration of cotton cellulose in [DIMIM][(MeO)(H)PO_2_] and [EMIM][(MeO)(H)PO_2_].

Cotton Concentration in the Solution (wt%)	[DIMIM][(MeO)(H)PO_2_]			[EMIM][(MeO)(H)PO_2_]		
	Weight Gain after Regeneration (%)	P Content Calculated from Weighting (wt%)	Phosphorus Content from ICP-AES) (wt%)	Weight Gain after Regeneration (%)	P Content Calculated from Weighting (wt%)	Phosphorus Content from ICP-AES (wt%)
0.5	36	5.1	4.51	/	/	/
1	26	4.0	/	20	3.0	/
2	25	3.9	3.23	/	/	/
5	18	2.9	2.82	14	2.2	1.29
10	19	3.1	2.02	16	2.4	1.31
